# Comparative evaluation of different debonding and reconditioning methods for orthodontic ceramic brackets regarding effectiveness for reuse: An in vitro study

**DOI:** 10.1007/s00056-023-00469-z

**Published:** 2023-06-15

**Authors:** Katharina Grosch, Jörg Meister, Sanjay D. Raval, Ahmed Mahmoud Fouda, Christoph Bourauel

**Affiliations:** 1 Department of Oral Technology, Center of Dentomaxillofacial Medicine, University Hospital Bonn, Bonn, Germany; 2 Department of Periodontology, Operative and Preventive Dentistry, Center of Dentomaxillofacial Medicine, University Hospital Bonn, Bonn, Germany; 3 Center of Applied Medical Laser Research and Biomedical Optics (AMLaReBO), University Hospital Bonn, Bonn, Germany; 4 Private practice, Bretten, Germany

**Keywords:** Reusability, Shear bond strength, Fixed orthodontic appliances, Er:YAG laser, Material properties, Wiederverwendbarkeit, Scherhaftfestigkeit, Festsitzende kieferorthopädische Apparaturen, Er:YAG-Laser, Materialeigenschaften

## Abstract

**Purpose:**

To investigate the reusability of ceramic brackets in terms of shear bond strength, friction behavior, slot dimension, fracture strength, and color stability.

**Methods:**

A total of 90 conventionally debonded and 30 by an Er:YAG laser debonded ceramic brackets were collected. All the used brackets were inspected under a stereomicroscope at 18 × magnification and sorted according to their adhesive remnant index (ARI). Five groups were formed (*n* = 10): (1) new brackets as a control group, (2) flamed and sandblasted, (3) flamed and acid bathed, (4) laser-reconditioned, and (5) laser-debonded brackets. The bracket groups were tested regarding different properties such as shear bond strength, friction behavior, slot size, fracture strength, and color stability. Analysis of variance (ANOVA) and nonparametric Kruskal–Wallis tests were used for statistical analysis (significance level: *p* < 0.05).

**Results:**

Shear bond strength values of the acid reconditioned brackets were significantly lower (8.0 ± 3.1 MPa) compared to the control group (12.9 ± 2.9 MPa). Laser-reconditioned (32.8 ± 2.7%) and laser-debonded (30.9 ± 2.4%) brackets showed the lowest force loss due to friction (control group 38.3 ± 3.0%). No significant differences were observed between groups regarding slot size and fracture strength. All groups had color differences of ${\Updelta E}_{ab}^{\mathrm{*}}$< 10. Scanning electron microscope images and ARI scores indicated that most of the residues on the bracket bases were removed.

**Conclusion:**

All reconditioning methods yielded adequate results regarding bracket properties. Yet, focusing on the need to protect the enamel and the bracket base, laser debonding seems to be the most suitable method for reconditioning ceramic brackets.

## Abstract (de)

**Zielsetzung:**

Untersuchung der Wiederverwendbarkeit keramischer Brackets in Bezug auf Scherhaftfestigkeit, Reibungsverhalten, Slotdimension, Bruchfestigkeit und Farbstabilität.

**Methoden:**

Insgesamt wurden 90 konventionell und 30 mit einem Er:YAG-Laser debondete Keramikbrackets gesammelt. Alle verwendeten Brackets wurden unter einem Stereomikroskop bei 18-facher Vergrößerung untersucht und nach ihrem ARI („adhesive remnant index“) sortiert. Es wurden 5 Gruppen gebildet: (1) neue Brackets als Kontrollgruppe, (2) geflammte und sandgestrahlte, (3) geflammte und säuregebadete, (4) Laser-aufbereitete sowie (5) Laser-debondete Brackets. Die Bracketgruppen wurden auf Scherhaftfestigkeit, Reibungsverhalten, Slotdimensionen, Bruchfestigkeit und Farbstabilität getestet. Jede Untersuchung wurde an 10 Brackets durchgeführt. Für die statistische Analyse wurden Varianzanalysen (ANOVA) und nichtparametrische Kruskal-Wallis-Tests verwendet (Signifikanzniveau: *p* < 0,05).

**Ergebnisse:**

Die Scherhaftfestigkeitswerte der säuregebadeten Brackets waren signifikant niedriger (8,0 ± 3,1 MPa) als die der Kontrollgruppe (12,9 ± 2,9 MPa). Laser-aufbereitete (32,8 ± 2,7 %) und Laser-debondete (30,9 ± 2,4 %) Brackets zeigten den geringsten Reibungsverlust (Kontrollgruppe 38,3 ± 3,0 %). Bei den Messungen von Slotdimension und Bruchfestigkeit wurden keine signifikanten Unterschiede festgestellt. Alle Gruppen hatten einen Farbunterschied von ${\Updelta E}_{ab}^{\mathrm{*}}$< 10. Rasterelektronenmikroskopische Bilder und ARI-Auswertungen zeigten, dass die meisten Klebereste in den Bracketbasen entfernt wurden.

**Schlussfolgerung:**

Mit allen Aufbereitungsmethoden konnten angemessene Ergebnisse hinsichtlich der Bracketeigenschaften erzielt werden. Unter Berücksichtigung der Notwendigkeit, den Zahnschmelz und die Bracketbasis zu schützen, scheint das Laser-Debonding die am besten geeignete Methode für das Aufbereiten von Keramikbrackets zu sein.

## Introduction

For adolescents and increasingly for adults, esthetic aspects are nowadays an important part of orthodontic treatment [1, 2]. In addition to lingual techniques and aligners, esthetic multibracket appliances continue to play an important role especially in the more visible anterior region.

Compared to aligners and other bracket materials, ceramic orthodontic brackets have the advantage of having high mechanical and chemical resistance, while providing the required properties and esthetics for modern orthodontics [3–6]. One major disadvantage is that ceramic brackets are significantly more expensive than polymer brackets and less esthetic metal brackets. To cope with this, reuse of ceramic brackets needs to be considered. Yet, according to the manufacturers, brackets are intended for single use only. Thus, very few brackets are reused in orthodontic everyday practice, most often in cases where a single bracket debonded and requires re-bonding. But, even in these cases, new brackets are frequently used. Usually, used brackets are discarded after treatment. There are several studies, however, indicating that reuse of ceramic brackets may be possible [7–9].

Efficient reconditioning of ceramic brackets could minimize the high treatment costs. This could make orthodontic treatment with esthetic alternatives more accessible to patients. Furthermore, the process of reconditioning ceramic brackets is relevant from a sustainability perspective in terms of a cradle-to-cradle (C2C) economy to conserve natural resources [10, 11].

To prevent microcracks from occurring in the enamel and the bracket’s susceptibility to fracture [4, 5, 12] during conventional debonding with pliers, alternative debonding procedures that protect the bracket as well as the enamel are in demand. In several studies it was demonstrated that debonding of ceramic brackets by laser is possible and protects the enamel since the adhesive remains on the enamel surface [7, 13–18]. This effect is promising when considering laser debonding as an efficient method to debond ceramic brackets.

However, there is little research showing how the quality of ceramic brackets changes after debonding with subsequent preparation for reuse. Property changes such as shear bond strength, fracture resistance, frictional behavior, and changes in slot dimension as well as possible color changes while removing and processing the brackets for reuse need to be further studied. Therefore, our research was dedicated to investigating bracket properties following common methods of bracket debonding and treatment.

## Materials and methods

### Sample preparation and group design

A total of 120 ceramic brackets (Damon® Clear™ 2, Ormco, Brea, CA, USA) were collected from an orthodontic office. After completion of orthodontic treatment, the brackets were debonded, roughly checked for damage and then sorted by bracket type (anterior, canine, posterior). All brackets were debonded by the same orthodontist in one private practice. Ninety brackets were conventionally debonded with a side cutter and 30 brackets were detached from teeth using the KaVo Key III Laser (Er:YAG laser, wavelength 2.94 μm, KaVo Dental GmbH, Biberach, Germany), which is approved for dental application. The average output power was 0.75 W and the laser was used with a pulse energy of 250 mJ and a pulse repetition rate of 3 Hz with air-cooling. The handpiece used was a noncontact handpiece (type 2060). No laser-debonded brackets showed microcracks after debonding, whereas 30% of the brackets were damaged during debonding with pliers and were sorted out immediately. All brackets were sterilized directly after debonding.

It is explicitly noted at this point that this is not a clinical study. Patients were treated as usual, and treatment was completed according to the usual orthodontic treatment criteria. Permission was only sought to collect the brackets anonymously for the scientific study. If permission was denied, the brackets were disposed as usual, otherwise they were added to the collection. Consent was recorded. The decision as to whether debonding was done conventionally with pliers or with the laser was made by the orthodontist in consultation with the patient. The only criteria in deciding whether to debond the brackets with the laser was the patient’s consent. Selection of patients was randomized.

The brackets were first subjected to careful inspection for visible damage under a stereomicroscope (Wild M8, Heerbrugg, Switzerland) at 18 × magnification. The conventionally sheared brackets were then sorted in a way that the three groups were obtained each with equal proportions of ARI 0–3 according to the adhesive remnant index (ARI) of Årtun and Bergland [19]:

- ARI 0 = all adhesive left on the bracket—no adhesive left on the tooth
- ARI 1 ≥ 50% of the adhesive left on the bracket—≤ 50% of the adhesive left on the tooth
- ARI 2 ≤ 50% of the adhesive left on the bracket—≥ 50% of the adhesive left on the tooth
- ARI 3 = no adhesive left on the bracket—all adhesive left on the tooth.

This was done to prevent a distorted evaluation of the efficiency of the reconditioning methods described below due to differing amounts of adhesive on the bracket base.

### Reconditioning methods

**Fig. 1:**
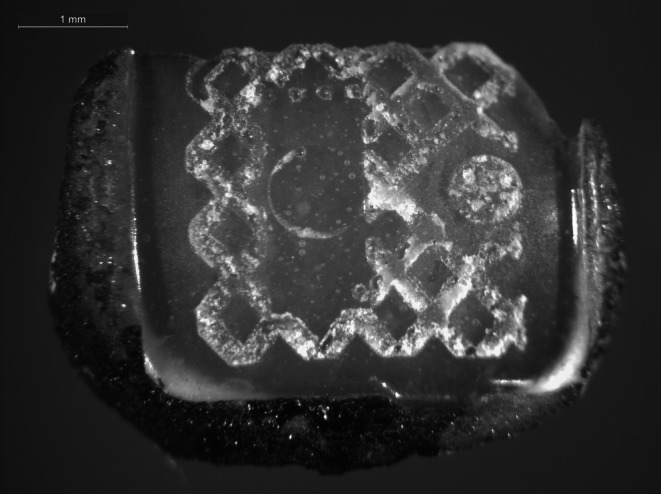
Detached adhesive from the bracket base due to fast cooling down after flaming. 18 × magnification Abgelöster Klebstoff von der Bracketbasis durch schnelles Abkühlen nach Abflämmen. Vergr. 18:1

Debonded brackets were divided into three groups according to the following reconditioning methods:

- In the sandblasting group, adhesive was first burned out of the bracket base with a torch (Buffalo Dental Manufacturing Co. Inc., Syosset, NY, USA). The bracket was held over the flame for 5 s and then immersed immediately in ethanol. This process was repeated up to 20 times. Due to the rapid cooling down of the material, the adhesive detached from the bracket base (Fig. 1). Residues were then removed by sandblasting using 50 μm lead-free sodium glass beads (Perlablast, BEGO, Bremen, Germany) with 5 mm distance between the bracket base and the hand piece head until no adhesive remnants could be detected [9]. Then, the bracket was rinsed with air spray for 10 s to remove any residual powder. The maximum total work time for reconditioning one bracket was 3 min.
- In the acid group, adhesive was first burned out of the bracket base, as described in the sandblasting group. Afterwards, remnants were removed using an acid (32% hydrochloric acid and 55% nitric acid in a 1:4 ratio) at temperatures of approximately 80 °C in an ultrasonic bath for 45 min. Following the acid bath, the brackets were placed under running water for 60 s [20]. The maximum total work time for reconditioning one bracket was 3 min.
- In the laser group, bracket bases were irradiated with the same Er:YAG laser used for debonding the brackets from the patient. The average output power was 6 W, and the laser was used at 400 mJ pulse energy and 15 Hz pulse repetition rate with air-cooling from 10 mm distance (noncontact handpiece 2060) for 10 s. The bracket was then immersed in ethanol. This process was repeated up to 20 times until no adhesive was visible to the naked eye. As described by Mirhashemi et al. [9], the bracket base was held perpendicular to the laser to remove adhesive during laser irradiation. Operators wore protective glasses during laser application. The maximum total work time for reconditioning one bracket was 4 min.

The abbreviations for the reconditioning methods used in this article are listed in Table 1.

**Table 1:** Abbreviations of experimental groups Abkürzungen für die Versuchsgruppen

Reconditioning method	Abbreviation
*N*ew brackets (control group)	DN
Flamed and *s*andblasted brackets	DS
Flamed and *a*cid bath brackets	DA
*L*aser-reconditioned brackets	DL
*L*aser-*d*ebonded brackets	DLD

*D* Damon

### Shear bond strength

The shear bond strength was investigated according to DIN 13990 [21]. Permanent bovine central incisors were selected in accordance with the test specifications of DIN 13990 to obtain a sufficiently large number and adequate quality of suitable enamel surfaces. The teeth were placed in round specimen holders, fixed with wax, and encapsulated with a self-curing acrylic resin (Technovit 4004; Kulzer GmbH, Hanau, Germany) so that the adhesive surface was aligned parallel to the shear direction when the specimen was mounted vertically in a materials testing machine. The brackets with applied adhesive were placed on the teeth [22]. The adhesive used was Transbond XT™ (3M Unitek, Monrovia, CA, USA). Preparation and measurements of test specimens were carried out strictly according to the DIN standard. A shear plate was placed under the bracket and shear load was applied. Measurements were carried out in a materials testing machine (Zwick ZMART.PRO, Zwick, Ulm, Germany) in cervicoincisal direction at a traverse speed of 1 mm/min.

### Force loss due to friction

**Fig. 2:**
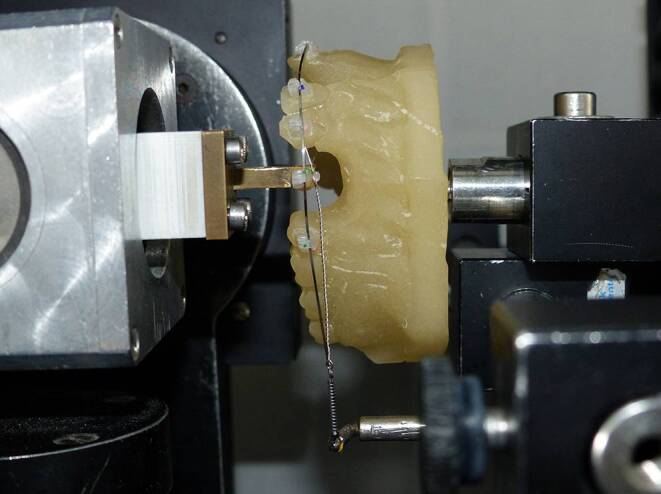
Mechanical test setup for measuring the force loss due to friction in the Orthodontic Measurement and Simulation System (OMSS). Test model with ligated arch wire and glued bracket to the force/torque sensor. Orthodontic force is transferred to the bracket by a NiTi tension spring Mechanischer Versuchsaufbau zur Messung des Kraftverlusts durch Reibung im orthodontischen Mess- und Simulationssystem (OMSS). Testmodell mit ligiertem Führungsbogen und am Kraft‑/Drehmomentsensor aufgeklebtem Bracket. Die Kraft wird durch eine NiTi-Zugfeder auf das Bracket übertragen

Measurements with the Orthodontic Measurement and Simulation System (OMSS) have been proven to be the closest at representing clinical tooth movement compared to other experimental methods [23, 24]. The OMSS was used to simulate canine retraction. A Frasaco model was equipped with the bracket system and a suitable archwire with the dimension of 0.46 × 0.64 mm^2^ (18 × 25, remanium®, Dentaurum, Ispringen, Germany) and ligated into the bracket slots. The examined bracket was then connected to the force/torque sensor of the OMSS. The force simulating the tooth movement was applied by a nickel–titanium (NiTi) tension coil spring similar to the clinical situation. The unit performs the canine retraction movement automatically, recording all the forces and torques acting on the bracket and spring. The experimental situation is shown in Fig. 2.

The force loss due to friction was calculated as the difference between the force applied by the NiTi tension spring and the effective force on the tooth. Each bracket was measured twice and the respective weighted averages were calculated.

### Slot size

**Fig. 3:**
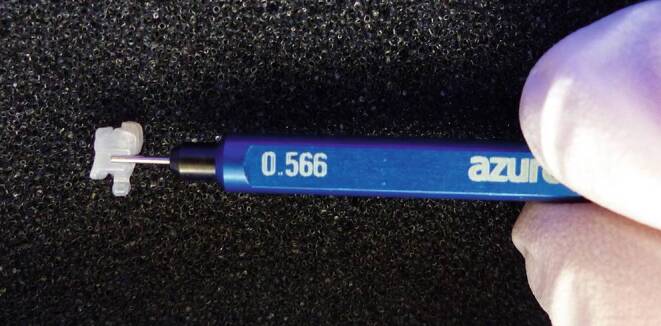
Measurement of the slot size using a pin gauge Messung der Slotgröße mit einem Lehrdorn

All brackets had a nominal slot size of 0.022 inch (0.56 mm). The slot dimensions of the canine brackets were determined with special pin gauges with rounded shapes (Azurea Jauges SA, Belprahon, Switzerland). These pin gauges are made of hardened steel and guarantee an accuracy of ±0.0004 mm. Slot sizes were measured at 0.002 mm intervals with the available pin gauges from 0.556 to 0.600 mm. The slot dimension was determined by inserting the pin gauges into the slot from the smallest in ascending order (Fig. 3). This method has been used in a similar way in previous experiments [25, 26]. The size of the last inserted pin gauge thus documented the slot dimension.

### Fracture strength

**Fig. 4:**
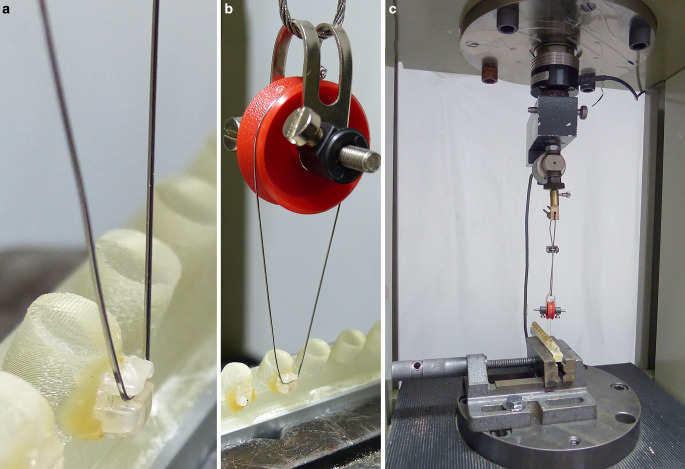
Experimental setup: **a** Bonded bracket on a specimen holder connected with a pulley on the force sensor mounted in the material testing machine. **b** The ligature wire was placed under the bracket wing. **c** Overall view of the experimental setup with specimens installed in the materials testing machine Versuchsaufbau: **a** geklebtes Bracket auf einem Probenhalter, der über eine Umlenkrolle mit dem Kraftsensor in der Materialprüfmaschine verbunden ist. **b** Der Ligaturendraht wurde unter dem Bracketflügel platziert. **c** Gesamtansicht des Versuchsaufbaus mit den in der Werkstoffprüfmaschine installierten Prüfkörpern

Neither standards nor test specifications exist for measuring the fracture strength of orthodontic ceramic brackets. Hence, the procedure described by Sanchez et al. [27] was used as a guideline. For this purpose, premolar brackets were fixed on a self-designed specimen holder with a two-component synthetic resin adhesive (Pattex Stabilit Express, Henkel, Düsseldorf, Germany) and mounted on the material testing machine (Zwick ZMART.PRO, Zwick, Ulm, Germany). The force sensor of the Zwick material testing machine was connected to a bracket wing via a ligature wire (diameter 0.40 mm, remanium®, Dentaurum, Ispringen, Germany). Then, tensional force was applied to the bracket wing at 10 mm/min traverse speed until fracture occurred. The experimental setup is displayed in Fig. 4. The fracture surfaces were examined under the microscope and photographed. Images were captured at 18 × magnification using a digital camera (DFC420 C, Leica, Wetzlar, Germany) attached to the stereomicroscope (Wild M8, Heerbrugg, Switzerland). The area was determined using an image analysis software (ImageJ Version 1.53 for macOS). The fracture strength was then calculated from the measured force and the determined fracture surface.

### Color stability

Generally accepted standards exist for the measurement of color differences which refer to the measurement of color distances in the CIE L*a*b* color space [28, 29]. After a calibration procedure, each bracket was measured six times with the spectrophotometer VITA Easyshade V® (VITA Zahnfabrik, Bad Säckingen, Germany) on a white background. The bracket was positioned in a template made from a 1 mm thick plastic plate (Copyplast, Scheu-Dental, Iserlohn, Germany) by thermoforming to ensure that the same position on the specimens were always selected for measurements. After each measurement, the color coordinates (L*, a*, b*) displayed by the device were recorded. L* stands for the brightness, a* for the color on the red–green axis, and b* for the color on the yellow–blue axis. The color difference (${\Updelta E}_{ab}^{\mathrm{*}}$) between the control group and the other groups was then calculated using the following formula [29]:

1 $${\Updelta E}_{ab}^{\mathrm{*}}=\sqrt{\left(\Updelta L^{\mathrm{*}}\right)^{2}+\left(\Updelta a^{\mathrm{*}}\right)^{2}+\left(\Updelta b^{\mathrm{*}}\right)^{2}}$$

### Adhesive remnant index

After debonding, the amount of adhesive remnants on each bracket surface was observed under a stereomicroscope (Wild M8, Heerbrugg, Switzerland) at 18 × magnification. This was repeated after the reconditioning process.

### Scanning electron microscope

One bracket was randomly selected from each group. The surface of each specimen was coated with a thin gold/platinum layer using a sputter coater (Scancoat six; Edwards High Vacuum, England, UK). The brackets were examined under the scanning electron microscope (SEM; Philips XL 30 CP, Philips, Eindhoven, The Netherlands) operated at 10 kV at 300 × magnification.

### Statistical analysis

Data were statistically analyzed using GraphPad Prism Version 9.4.1 for macOS. The Shapiro–Wilk test was used to determine whether the variables followed a normal distribution. Since shear bond strength, force loss due to friction, changes in slot dimension, color parameters (L*, a*, b*) and fracture strength were normally distributed, one-way analysis of variance (ANOVA) with a subsequent Bonferroni test was applied. The nonparametric Kruskal–Wallis test was applied for ARI values and post hoc comparisons were made with Dunn’s test. The significance level was set at *p* < 0.05. After Bonferroni and Dunn’s corrections, adjusted *p*-values were used to determine significance. For ${\Updelta E}_{ab}^{\mathrm{*}}$, the Gaussian error propagation was applied, and confidence intervals were calculated, as ${\Updelta E}_{ab}^{\mathrm{*}}$ is described by a composite equation, depending on three variables.

## Results

### Shear bond strength

The mean shear bond strength, the corresponding standard deviation of each group and the associated *p*-values compared to the new brackets are shown in Table 2. Reconditioned brackets after laser debonding showed similar mean shear bond strength values (12.8 ± 2.8 MPa) compared to the new brackets (12.9 ± 2.9 MPa). No statistically significant difference was observed between the two groups (*p* > 0.99). The mean shear bond strength of the new brackets was 12.9 ± 2.9 MPa. The only significant difference compared to the new brackets was observed in the acid group (8.0 ± 3.1 MPa, *p* = 0.002).

**Table 2:** Mean, standard deviation, and statistical analysis of the shear bond strength in MPa Mittelwert, Standardabweichung und statistische Auswertung der Scherhaftfestigkeit in MPa

Group	*n*	Mean (MPa)	SD	*p*
DN	10	12.9	2.9	–
DS	10	9.8	2.1	0.09
DA	10	8.0	3.1	0.002*
DL	10	10.8	3.4	0.41
DLD	10	12.8	2.8	> 0.99

*SD* Standard deviation. See Table 1 for other abbreviations

*p < 0.05 versus the control group

### Force loss due to friction

**Fig. 5:**
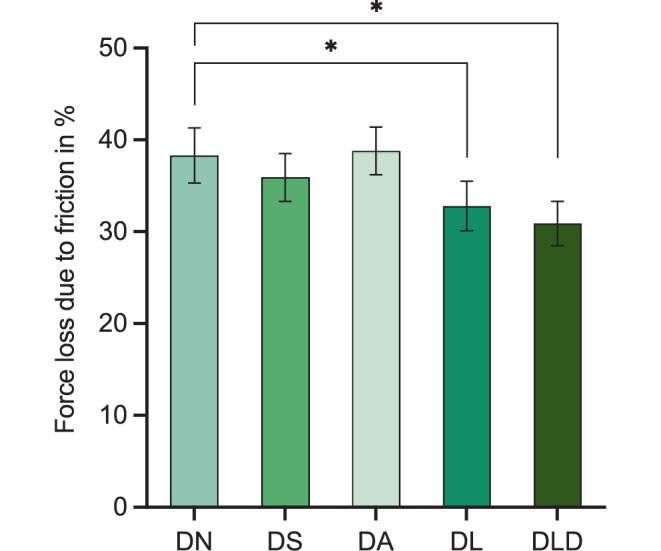
Force loss due to friction expressed as a percentage of the applied force on the brackets. *Asterisks* show a significant difference between groups (*p* < 0.05). See Table 1 for abbreviations Kraftverlust aufgrund von Reibung, als Prozentsatz der aufgebrachten Kraft auf die Brackets. *Sternchen* zeigen einen signifikanten Unterschied zwischen den Gruppen (*p* < 0,05). Abkürzungen siehe Tab. 1

The results of the force loss due to friction are visualized in Fig. 5. The Bonferroni post hoc test indicated a significantly smaller force loss due to friction in the laser-reconditioned (32.8 ± 2.7%, *p* < 0.001) and laser-debonded (30.9 ± 2.4%, *p* < 0.001) groups compared to the control group (38.3 ± 3.0%). On the contrary, there was no statistically significant difference between the sandblasted (35.9 ± 2.6%) and acid-treated brackets (38.8 ± 2.6%) compared to new brackets.

### Slot size

**Fig. 6:**
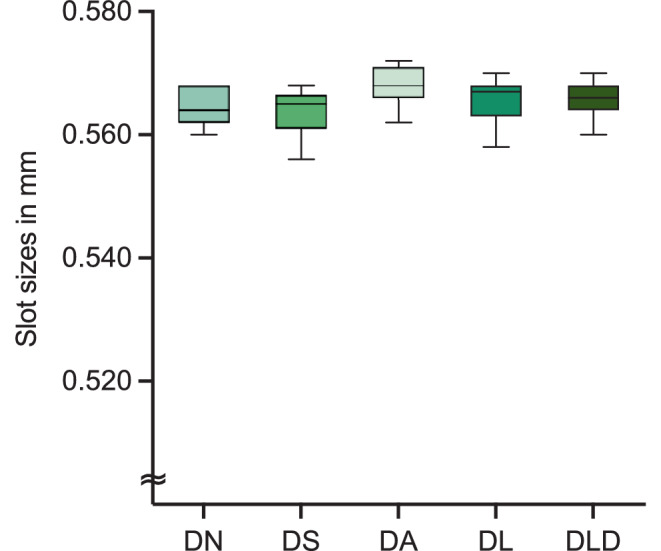
Box and whisker plot illustrating the measured slot sizes in mm. See Table 1 for abbreviations Box-und-Whisker-Diagramm zur Darstellung der gemessenen Slotgröße in mm. Abkürzungen siehe Tab. 1

The measured slot dimensions are shown in Fig. 6. None of the measured slot sizes were beyond the tolerance range of 0.560 + 0.040 mm according to DIN 13996 [30]. Furthermore, there was no significant difference between any of the reconditioned brackets and the new brackets (0.565 ± 0.003 mm,* p* > 0.05). Brackets after treatment showed slot sizes from 0.564 ± 0.004 mm to 0.568 ± 0.003 mm.

### Fracture strength

**Fig. 7:**
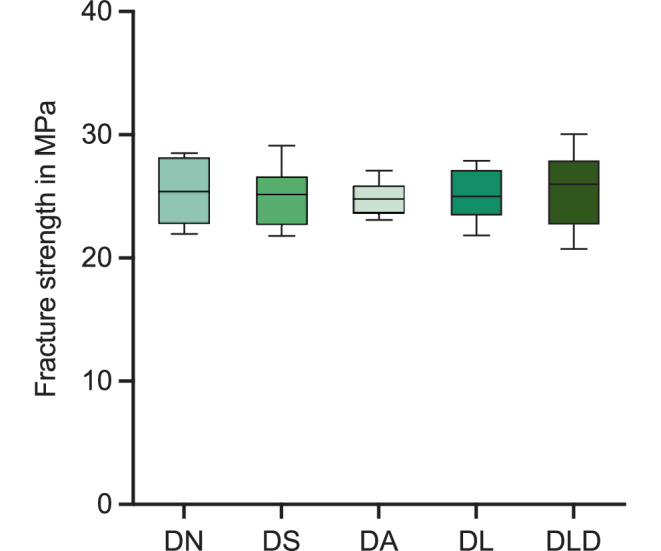
Box and whisker plot illustrating the fracture strength in MPa. See Table 1 for abbreviations Box-und-Whisker-Diagramm zur Darstellung der gemessenen Bruchfestigkeit in MPa. Abkürzungen siehe Tab. 1

The determined fracture strengths of all brackets ranged between approximately 20 and 30 MPa. Figure 7 depicts the results. Bonferroni’s post hoc test showed no significant differences between the groups (*p* > 0.99).

### Color stability

The mean values, the corresponding standard deviations of each group, and the associated *p*-values compared to the new brackets are shown in Table 3. The sandblasting, laser reconditioning and laser debonding groups had significantly lower L* (brightness) values (*p* < 0.05) than the new brackets. The mean a* (color on the red–green axis) value of the acid group was significantly lower than that of the control group (*p* = 0.003). Within the b* (color on the yellow–blue axis) values, significant differences were found between all groups (*p* < 0.05), except for the acid group compared to the control group (*p* > 0.05). The conventionally debonded brackets showed a ${\Updelta E}_{ab}^{\mathrm{*}}$ between 6.3 and 7.4 before treatment. The acid group showed the lowest ${\Updelta E}_{ab}^{\mathrm{*}}$ of 2.9 ± 1.1 followed by the sandblasting group (6.5 ± 1.3). The laser-debonded group (9.4 ± 0.6) and the laser-reconditioned group (9.8 ± 1.8) showed the highest ${\Updelta E}_{ab}^{\mathrm{*}}$. The analysis of the confidence intervals indicated that the acid group had a significantly lower ${\Updelta E}_{ab}^{\mathrm{*}}$ compared to the other groups. The laser-reconditioned and the laser-debonded groups were the only groups showing a significant increase of ${\Updelta E}_{ab}^{\mathrm{*}}$ compared to the pretreatment value. Furthermore, the laser-reconditioned and the laser-debonded groups had a significantly higher ${\Updelta E}_{ab}^{\mathrm{*}}$ than the sandblasted group.

**Table 3:** Mean, standard deviation, statistical analysis of the color parameters L*, a*, b*, ${\Updelta E}_{\mathrm{ab}}^{\mathrm{*}}$ and 95% confidence intervals (CI) for ${\Updelta E}_{\mathrm{ab}}^{\mathrm{*}}$ Mittelwert, Standardabweichung, statistische Auswertung der Farbparameter L*, a*, b*, ${\Updelta E}_{\mathrm{ab}}^{\mathrm{*}}$ und 95%-Konfidenzintervalle für ${\Updelta E}_{\mathrm{ab}}^{\mathrm{*}}$

	L*	*p *(L*)	a*	*p *(a*)	b*	*p *(b*)	${\Updelta E}_{\mathrm{ab}}^{\mathrm{*}}$	Lower 95% CI	Upper 95% CI
DN	74.6 ± 2.8	–	0.3 ± 0.2	–	2.4 ± 0.4	–	–	–	–
DS bt	70.1 ± 2.5	0.06	0.5 ± 0.5	> 0.99	8.3 ± 3.7	< 0.001*	7.4 ± 2.6	5.5	9.4
DS	69.9 ± 3.2	0.04*	0.3 ± 0.3	> 0.99	6.9 ± 2.2	< 0.001*	6.5 ± 1.3	5.5	7.5
DA bt	70.8 ± 6.3	0.18	0.5 ± 0.4	> 0.99	7.2 ± 2.0	< 0.001*	7.0 ± 3.5	4.4	9.6
DA	76.3 ± 3.9	> 0.99	−0.2 ± 0.3	0.003*	4.7 ± 1.5	0.15	2.9 ± 1.1	2.1	3.7
DL bt	70.5 ± 4.6	0.11	0.4 ± 0.4	> 0.99	7.2 ± 2.1	< 0.001*	6.3 ± 1.7	5.1	7.7
DL	66.9 ± 2.4	< 0.001*	0.7 ± 0.3	0.11	8.4 ± 3.3	< 0.001*	9.8 ± 1.8	8.4	11.1
DLD	67.8 ± 2.3	< 0.001*	0.5 ± 0.4	> 0.99	8.8 ± 1.1	< 0.001*	9.4 ± 0.6	8.9	9.8

*bt* before treatment. See Table 1 for other abbreviations

*p < 0.05 versus the control group

### Adhesive remnant index

Adhesive remnant index (ARI) scores are shown in Table 4. After the reconditioning treatments, most samples had an ARI score of 3. The data of the Bonferroni post hoc comparison test showed statistically significant differences in the ARI score only between the acid group compared to new brackets (*p* < 0.001).

**Table 4:** Frequency distribution of adhesive remnant index (ARI) values, ARI scores, and statistical analysis of the investigated groups Häufigkeitsverteilung des ARI („adhesive remnant index“), ARI-Werte und statistische Auswertung der untersuchten Gruppen

Group	*n*	0	1	2	3	Total	*p*
DN	30	0	0	0	30	3.00	–
DS bt	30	5	7	14	4	1.57	–
DS	30	0	0	1	29	2.97	> 0.99
DA bt	30	5	7	13	5	1.60	–
DA	30	0	0	14	16	2.53	< 0.001*
DL bt	30	6	6	13	5	1.57	–
DL	30	0	0	5	25	2.83	0.32
DLD	30	0	0	4	26	2.87	0.64

*bt* before treatment. See Table 1 for other abbreviations

*𝑝 < 0.05 versus the control group

### Scanning electron microscope

**Fig. 8:**
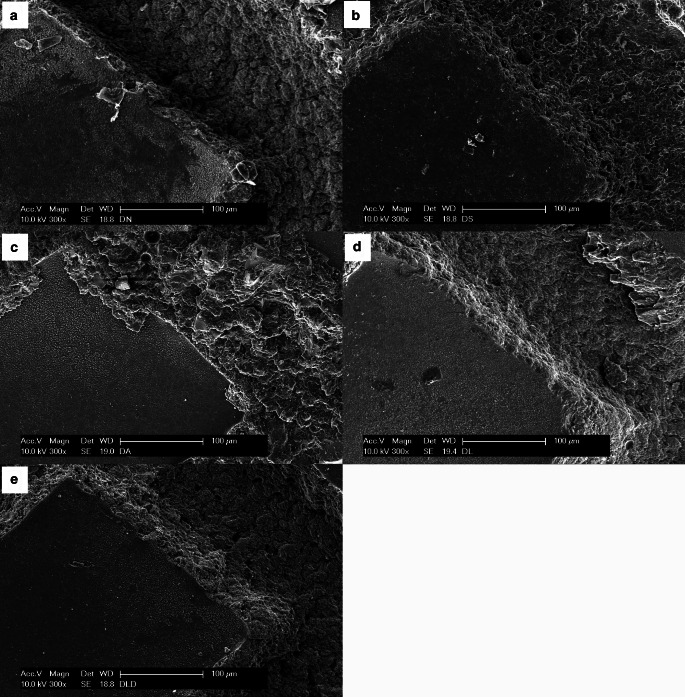
Scanning electron microscope images showing the bracket bases at 300 × magnification. **a** DN **b** DS **c** DA **d** DL **e** DLD. See Table 1 for abbreviations Rasterelektronenmikroskopische Aufnahmen der Bracketbasis, Vergr. 300:1. **a** DN **b** DS **c** DA **d** DL **e** DLD. Abkürzungen siehe Tab. 1

The scanning electron microscope (SEM) images (Fig. 8) showed differences between the reconditioning methods. Even though no adhesive residue was visible in the photo of the sandblasted bracket, erosion at the base of the bracket was visible (Fig. 8b). The acid-reconditioned brackets were the only group with adhesive left on the base (Fig. 8c). The bracket base from the laser-reconditioned and laser-debonded brackets appeared as clean as the new bracket base and showed only few signs of erosion (Fig. 8a, d, and e).

## Discussion

The purpose of this study was to examine the possibility of reusing ceramic brackets without decisively changing their characteristics for clinical usage. Reuse of brackets should become a state-of-the-art method in modern orthodontics, given the increasing need for saving our resources.

Studying shear bond strength after reconditioning is important since it has a direct impact on the resistance to functional and orthodontic forces. Especially resistance to chewing forces, and the force required upon debonding can lead to enamel damage because of high shear bond strength [5, 12]. None of the considered reconditioning methods except for the acid treatment resulted in brackets showing any significant differences in shear bond strength compared to new brackets (Table 2). The reduced shear bond strength of acid-treated brackets can also be correlated with the lower ARI score. The acid group was the only significantly different group with an averaged ARI score of 2.53 after treatment compared to new brackets (Table 4). The SEM images confirmed that acid treatment was not able to remove all the adhesive from the bracket base (Fig. 8c). Since the adhesive residues filled the intended laser etched pattern for creating retention surfaces in the bracket base, the new adhesive was not able to build up the necessary adhesive forces. Further adhesion processes were thereby impaired.

Sandblasting showed no decrease of the shear bond strength, as was previously reported by Yassaei et al. [7] and Mirhashemi et al. [9]. However, the SEM images (Fig. 8b) showed that sandblasting eroded the structures of the bracket base resulting in a small but not significant decrease in shear bond strength. Furthermore, no adhesive residues were seen in the SEM images, which verifies the high ARI score of 2.97 (Table 4). Overall, these results support the conclusion of Khanal et al. [31] that the shear bond strength of sandblasted brackets is in accordance with clinical requirements. A significantly lower shear bond strength as shown by Han et al. [8] and Urichianu et al. [32] compared to new brackets was not seen in this study. The difference in shear bond strength of the described studies regarding sandblasting treatments could be caused by varied duration of sandblasting, different sandblasting media, and different distances from the bracket base, which could lead to different amounts of erosion.

The mean shear bond strength and average ARI scores of laser-reconditioned brackets did not significantly differ from new brackets. SEM images showed that the bracket bases were not at all or only slightly eroded (Fig. 8d). This supports the conclusion of Han et al. [8] that the microcrystalline structures of the brackets were not destroyed due to the laser treatment and complete removal of adhesive is possible. Research by Yassaei et al. [7] and Mirhashemi et al. [9] showed that laser treatment was able to remove the adhesive without harming the bracket or significantly reducing its shear bond strength, which is consistent with the present study results.

Laser-debonded brackets showed almost no adhesive remnants on the bracket base. Laser debonding resulted in a failure of the bond between the bracket and the adhesive. Therefore, the brackets could be reused without further treatments of the bracket base since almost all brackets showed an ARI of 3. Several previous studies [14] showed that the adhesive stayed on the enamel and that the enamel did not become damaged. Hence, the laser debonding method protects the bracket as well as the enamel. Furthermore, the SEM analysis showed only little to no visible erosion of the bracket base (Fig. 8e). As a result, shear bond strength of the laser-debonded brackets was almost identical to that of new brackets (*p* > 0.99).

Reliable comparative results are missing since no data about research on shear bond strength of laser-debonded brackets have been published yet. It can be derived from our present results that multiple reuses should be possible after treatment and laser debonding.

The evaluation and comparison of friction results is complicated since friction is an interaction of many effects. For example, Reimann et al. [33] stated that friction behavior depends on the material used for the arch wire and the ligature (see also El-Bialy et al. [34]), the shape and the roughness of the bracket slot (see also Schumacher et al. [35]) or the bracket material. Chang et al. [36] and Schumacher et al. [35] suggested that friction increases when the bracket bevel is smaller and decreases when the bracket bevel is larger. El-Bialy et al. [34] implied that a larger slot size decreased friction. For this reason, the results of our study cannot be easily compared to the results of other studies since not all influencing factors were observed. As opposed to Reimann et al. [37] who studied frictional behavior of reconditioned metal brackets, the results of our study showed a significant reduction of force loss due to friction of laser-debonded and -treated brackets compared to new brackets (Fig. 5). It is suspected that new brackets have imperfections due to the manufacturing process. During orthodontic use, these imperfections might be reduced when the wire slides through the slot. It can be assumed, regarding the present results, that sandblasting and acid treatment roughens the surface and thus the values do not differ significantly from new brackets, whereas laser treatment and laser deboning have no influence on surface roughness. For further insights, bracket slot needs to be further examined under SEM. Yet, the measured force losses due to friction with approximately 30–40% were consistent to the results documented by Szczupakowski [38].

The standard for dimensions and tolerances of orthodontic brackets is defined in DIN 13996 [30]. According to this standard, a 0.022 inch slot should have a slot width between 0.56 and 0.60 mm. In our study, all brackets met these requirements with an average slot size of 0.564–0.568 mm. According to various studies of the slot sizes of new brackets, the accuracy of the slot width varies considerably and often deviates from the specified size [25, 39–41]. This indicates that the slot size after the reconditioning process is not only dependent on the impact of the reconditioning process but also on a certain discrepancy from the new bracket. Nevertheless, the present study did not show any significant change in slot size compared to new brackets. This result corresponds with the results of Martina et al. [42]. From this point of view, reuse can be considered.

As mentioned in the “Materials and methods” section, since there are no standards for measuring fracture strength, the experimental setup explained by Sanchez et al. [27] and Johnson et al. [43] was followed as a guideline. However, the calculation of the fracture strength was changed. Sanchez et al. [27] and Johnson et al. [43] used the following formula to calculate fracture strength:

2 $$\sigma =\frac{\textit{fracture force in}\ N}{\textit{contact area between wire and tie-wing in mm}^{2}}$$

Since this formula seemed not comprehensible, the following formula [44] was used providing a more reliable and reproducible result:

3 $$\sigma =\frac{\textit{fracture force in}\ N}{\textit{fracture surface in mm}^{2}}$$

The results from our measurements were found to be in the upper range reported by Lindauer et al. [45]. This deviation might be caused by differences in testing speed or improvements in the ceramic materials over time. Overall, no significant difference in fracture strength was measured between the various groups (Fig. 7). This indicates that reuse of brackets is not limited by a reduction in fracture strength.

As shown in previous in vitro studies, coffee, tea, patients’ diets, and saliva led to discoloring of ceramic brackets [46–49]. Thus, this may explain the prediscoloration of the examined brackets compared to the new brackets in our study. In a pilot study dealing with the removal of the adhesive, flaming resulted in the brackets turning black. Consequently, the heating period was reduced as much as possible to prevent the bracket from turning black due to oxidation of the NiTi-pin in the self-ligating clip. It was observed that submerging the bracket directly into alcohol reduced the discoloring effect due to fast cooling. The sandblasted, laser-reconditioned, and laser-debonded brackets were significantly darker since they had a lower L* value. It can be assumed that the induced heat darkened the brackets, whereas the acid bath whitened the brackets. Moreover, the acid bath significantly lowered the a* value leading to a greener perception. However, the acid-treated brackets were the only group not having a significantly higher b* value. All other groups turned more yellowish.

Overall, brackets in the laser-reconditioned group were discolored the greatest with a ${\Updelta E}_{ab}^{\mathrm{*}}$ of 9.8 (± 1.8) compared to new brackets. It is discussed controversially, up to which ${\Updelta E}_{ab}^{\mathrm{*}}$ the discoloring is clinically acceptable. Many researchers suggest that a ${\Updelta E}_{ab}^{\mathrm{*}}$ below 3.3 or 3.7 is clinically acceptable [47, 50]. Others suggest that a higher ${\Updelta E}_{ab}^{\mathrm{*}}$ value is clinically acceptable since the perceived color of the brackets depends on the lighting color and other conditions [51]. Values of 5 < ${\Updelta E}_{ab}^{\mathrm{*}}$< 10 are perceived but can be considered acceptable. Subjectively, however, the limit value of ${\Updelta E}_{ab}^{\mathrm{*}}$ =10 can be considered a good basis for all brackets under investigation [52]. In our case, the discoloring effect is barely recognized by the human eye. As a result, we can support the claim that a ${\Updelta E}_{ab}^{\mathrm{*}}$< 10 is clinically acceptable. Moreover, the difference of the measurements when comparing digital methods or spectrophotometers must be considered [51]. Furthermore, a ${\Updelta E}_{ab}^{\mathrm{*}}$< 10 is less than the discoloring seen by drinking coffee or other beverages [46, 49, 52]. Acid reconditioning left the most residues on the base; however, acid reconditioning led to the best results regarding the bracket color. If and how acid treatment can be used to avoid strong discoloring of brackets should be investigated in further studies.

It has to be clearly stated that ceramic brackets from only one brand (Ormco, Brea, CA, USA) were investigated, which means that the results cannot be transferred without restriction to other manufacturers. Furthermore, it is important to mention that the suitability of reusing ceramic brackets was investigated in vitro only. In the clinical situation there are many influencing factors, and investigation is thus complex.

One issue that was not investigated in this study is the reuse of the brackets regarding bacterial contamination. Multiple studies show that sterilization of orthodontic products is possible [53–55]. However, the studies focused on new brackets rather than reused ones. Ardeshna et al. [54] showed that new brackets were already contaminated by various bacteria and concluded that new brackets should be sterilized before use. Legal aspects on sterilization are to be clarified and the effectiveness of sterilization of reconditioned brackets needs to be investigated in further studies.

Another aspect that needs to be investigated for practical application is economic efficiency. To calculate the costs of reconditioning, the duration of the individual reconditioning processes and costs of working time are multiplied. It must be noted, however, that professional reconditioning of large quantities of brackets would reduce the time needed for a single bracket. It is explicitly noted that this study does not aim to calculate an exact economic benefit of reconditioning. One major advantage for the patient could be that this esthetic treatment becomes more affordable and, thus, available to a wider range of patients presuming bracket manufacturers approve of the process. Regarding the conservation of resources and the complex manufacturing process, the potential of reusing high-quality ceramic brackets should be considered.

## Conclusions

Within the limitations of this study, it can be concluded that all methods of reconditioning yielded clinically acceptable results. For environmental and economic reasons, it is appropriate to integrate reconditioning methods into clinical practice. Reconditioned brackets showed no significant difference in slot dimensions and fracture strength. Compared to the new brackets, the shear bond strength was only significantly lower in the acid group. Most of the reconditioned brackets in all groups had an ARI score of 3. The aim of reconditioning ceramic brackets is to preserve an intact bracket base, while removing all adhesive from the base. This was only achieved by laser treatment and by laser debonding. Moreover, laser-treated and laser-debonded brackets provided significantly lower friction in the slot compared to new brackets, possibly making them more suitable for clinical use. Overall, laser debonding is considered the best method for reconditioning ceramic brackets in terms of the possibility of multiple reuses, and also the protection of enamel and the bracket. In hospitals and practices where an Er:YAG laser is available, it can be used for laser debonding and in cases where a bracket comes off, laser treatment could be used to remove the adhesive and rebond the bracket.

## References

[1] Singh S, Singla L, Anand T (2021) Esthetic considerations in orthodontics: an overview. Dent J Adv Stud 9:55–60. 10.1055/S-0041-1726473

[2] Xavier J, Sarika K, Ghosh P, Varma S (2021) Aesthetic bracket system: a review. Int J Dent Oral Sci 8:5191–5196. 10.19070/2377-8075-210001041

[3] Gautam P, Valiathan A (2007) Ceramic brackets: in search of an ideal! Trends Biomater Artif Organs 20:117–122

[4] Karamouzos A, Athanasiou AE, Papadopoulos MA (1997) Clinical characteristics and properties of ceramic brackets: a comprehensive review. Am J Orthod Dentofacial Orthop 112:34–40. 10.1016/S0889-5406(97)70271-3 9228839 10.1016/s0889-5406(97)70271-3

[5] Russell JS (2005) Aesthetic orthodontic brackets. J Orthod 32:146–163. 10.1179/146531205225021024 15994990 10.1179/146531205225021024

[6] Birnie D (1990) Ceramic brackets. Br J Orthod 17:71–75. 10.1179/BJO.17.1.71 2178681 10.1179/bjo.17.1.71

[7] Yassaei S, Aghili H, Firouzabadi AH, Meshkani H (2017) Effect of Er:YAG laser and sandblasting in recycling of ceramic brackets. J Lasers Med Sci 8:17–21. 10.15171/JLMS.2017.04 28912939 10.15171/jlms.2017.04 PMC5420360

[8] Han RQ, Yang K, Ji LF, Ling C (2016) Analysis of shear bond strength and morphology of Er:YAG laser-recycled ceramic orthodontic brackets. Biomed Res Int. 10.1155/2016/7276287 27047964 10.1155/2016/7276287 PMC4800079

[9] Mirhashemi AH, Hosseini MH, Chiniforoush N et al (2018) Shear bond strength of rebonded ceramic brackets using four different methods of adhesive removal. J Dent (Tehran) 15:54–62 29971122 PMC6026106

[10] Sherratt A (2013) Cradle to cradle. In: Idowu SO, Capaldi N, Zu L, Das Gupta A (eds) Encyclopedia of corporate social responsibility. Springer, Berlin, Heidelberg, pp 630–638

[11] McDonough W, Braungart M (2002) Cradle to cradle: remaking the way we make things, 1st edn. North Point Press, New York

[12] Swartz ML (1988) Ceramic brackets. J Clin Orthod 22:82–88 3075208

[13] Mundethu AR, Gutknecht N, Franzen R (2014) Rapid debonding of polycrystalline ceramic orthodontic brackets with an Er:YAG laser: an in vitro study. Lasers Med Sci 29:1551–1556. 10.1007/S10103-013-1274-9 23525867 10.1007/s10103-013-1274-9

[14] Oztoprak MO, Nalbantgil D, Erdem AS et al (2010) Debonding of ceramic brackets by a new scanning laser method. Am J Orthod Dentofacial Orthop 138:195–200. 10.1016/J.AJODO.2009.06.024 20691361 10.1016/j.ajodo.2009.06.024

[15] Ahrari F, Fekrazad R, Kalhori KAM, Ramtin M (2013) Reconditioning of ceramic orthodontic brackets with an Er,Cr:YSGG laser. Lasers Med Sci 28:223–228. 10.1007/S10103-012-1093-4 22585379 10.1007/s10103-012-1093-4

[16] Grzech-Leśniak K, Matys J, Zmuda-Stawowiak D et al (2018) Er:YAG laser for metal and ceramic bracket debonding: an in vitro study on intrapulpal temperature, SEM, and EDS analysis. Photomed Laser Surg 36:595–600. 10.1089/pho.2017.4412 29905504 10.1089/pho.2017.4412

[17] Khalil AS, Tamish NM, Elkalza AR (2022) Assessment of chemical, ultrasonic, diode laser, and Er:YAG laser application on debonding of ceramic brackets. BMC Oral Health. 10.1186/S12903-022-02111-7 35305631 10.1186/s12903-022-02111-7 PMC8933975

[18] Nalbantgil D, Oztoprak MO, Tozlu M, Arun T (2011) Effects of different application durations of ER:YAG laser on intrapulpal temperature change during debonding. Lasers Med Sci 26:735–740. 10.1007/S10103-010-0796-7 20535517 10.1007/s10103-010-0796-7

[19] Årtun J, Bergland S (1984) Clinical trials with crystal growth conditioning as an alternative to acid-etch enamel pretreatment. Am J Orthod 85:333–340. 10.1016/0002-9416(84)90190-8 6231863 10.1016/0002-9416(84)90190-8

[20] Dawjee S, Gheevarghese O (2004) Recycling debonded brackets with an acid bath. J Clin Orthod 38:605–606 15665432

[21] Deutsches Institut für Normung e. V (2017) DIN 13990:2017-04 Zahnheilkunde – Prüfverfahren für die Scherhaftfestigkeit von Adhäsiven für kieferorthopädische Befestigungselemente. Beuth-Verlag, Berlin

[22] Zielinski V, Reimann S, Jäger A, Bourauel C (2014) Comparison of shear bond strength of plastic and ceramic brackets. J Orofac Orthop 75:345–357. 10.1007/S00056-014-0236-6 25158948 10.1007/s00056-014-0236-6

[23] Bourauel C, Drescher D, Thier M (1992) An experimental apparatus for the simulation of three-dimensional movements in orthodontics. J Biomed Eng 14:371–378. 10.1016/0141-5425(92)90081-U 1405553 10.1016/0141-5425(92)90081-u

[24] Drescher D, Bourauel C, Thier M (1991) Orthodontisches Meß- und Simulationssystem (OMSS) für die statische und dynamische Analyse der Zahnbewegung. Fortschr Kieferorthop 52:133–140. 10.1007/BF02173245 1894242 10.1007/BF02173245

[25] Demling A, Dittmer MP, Schwestka-Polly R (2009) Comparative analysis of slot dimension in lingual bracket systems. Head Face Med. 10.1186/1746-160X-5-27 20003510 10.1186/1746-160X-5-27 PMC2803453

[26] Daratsianos N, Bourauel C, Flimmers R et al (2016) In vitro biomechanical analysis of torque capabilities of various 0.018″ lingual bracket-wire systems: total torque play and slot size. Eur J Orthod 38:459–469. 10.1093/ejo/cjv063 26518759 10.1093/ejo/cjv063

[27] Sanchez DJ, Walker MP, Kula K et al (2008) Fluoride prophylactic agents effect on ceramic bracket tie-wing fracture strength. Angle Orthod 78:524–530. 10.2319/052707-250.1 18416629 10.2319/052707-250.1

[28] Deutsches Institut für Normung e. V. (2017) DIN 5033-1:2017-10 Farbmessung – Teil 1: Grundbegriffe der Farbmetrik. Beuth-Verlag, Berlin

[29] Deutsches Institut für Normung e. V. (2020) DIN 11664-4:2020-03 Farbmetrik – Teil 4: CIE 1976 L*a*b* Farbraum (ISO/CIE 11664-4:2019). Beuth-Verlag, Berlin

[30] Deutsches Institut für Normung e. V. (2012) DIN 13996:2012-08 Zahnheilkunde – Maße für Drähte und Befestigungselemente für kieferorthopädische Anwendungen. Beuth-Verlag, Berlin

[31] Khanal PP, Shrestha BK, Yadav R, Gupta SP (2021) A comparative study on the effect of different methods of recycling orthodontic brackets on shear bond strength. Int J Dent. 10.1155/2021/8844085 33542734 10.1155/2021/8844085 PMC7843174

[32] Urichianu M, Makowka S, Covell D et al (2022) Shear bond strength and bracket base morphology of new and rebonded orthodontic ceramic brackets. materials. 10.3390/MA15051865 35269097 10.3390/ma15051865 PMC8911633

[33] Reimann S, Bourauel C, Weber A et al (2016) Friction behavior of ceramic injection-molded (CIM) brackets. J Orofac Orthop 77:262–271. 10.1007/S00056-016-0030-8 27142040 10.1007/s00056-016-0030-8

[34] El-Bialy T, Alobeid A, Dirk C et al (2019) Comparison of force loss due to friction of different wire sizes and materials in conventional and new self-ligating orthodontic brackets during simulated canine retraction. J Orofac Orthop 80:68–78. 10.1007/S00056-019-00168-8 30758513 10.1007/s00056-019-00168-8

[35] Schumacher H‑A, Bourauel C, Drescher D (1990) Das Friktionsverhalten von Keramikbrackets bei der bogengeführten Zahnbewegung. Fortschr Kieferorthop 51:259–265. 10.1007/BF02168926 2262178 10.1007/BF02168926

[36] Chang C‑J, Lee T‑M, Liu J‑K (2013) Effect of bracket bevel design and oral environmental factors on frictional resistance. Angle Orthod 83:956–965. 10.2319/101612-808.1 23621527 10.2319/101612-808.1 PMC8722825

[37] Reimann S, Rewari A, Keilig L et al (2012) Material testing of reconditioned orthodontic brackets. J Orofac Orthop 73:454–466. 10.1007/S00056-012-0108-X 23096945 10.1007/s00056-012-0108-x

[38] Szczupakowski AM (2015) Materialtechnische Untersuchungen an selbstligierenden und konventionellen Brackets mit verschiedenen Ligatursystemen zu ihrem tribologischen Verhalten. Rheinische Friedrich-Wilhelms-Universität, Bonn

[39] Divya P, Banswada SR, Kukunuru SR et al (2021) To compare the accuracy of 0.022 inch slot of stainless steel and ceramic orthodontic brackets marketed by different manufacturers. J Pharm Bioallied Sci 13:1037–1041. 10.4103/jpbs.jpbs_295_21 10.4103/jpbs.jpbs_295_21 PMC8687023 35017925

[40] Lefebvre C, Saadaoui H, Olive J‑M et al (2019) Variability of slot size in orthodontic brackets. Clin Exp Dent Res 5:528–533. 10.1002/cre2.219 31687187 10.1002/cre2.219 PMC6820806

[41] Hodecker L, Bourauel C, Braumann B et al (2023) Sliding behaviour and surface quality after static air polishing of conventional and modern bracket materials: in vitro analysis. J Orofac Orthop 84:110–124. 10.1007/s00056-021-00352-9 34554279 10.1007/s00056-021-00352-9 PMC9958151

[42] Martina R, Laino A, Cacciafesta V, Cantiello P (1997) Recycling effects on ceramic brackets: a dimensional, weight and shear bond strength analysis. Eur J Orthod 19:629–636. 10.1093/EJO/19.6.629 9458596 10.1093/ejo/19.6.629

[43] Johnson G, Walker MP, Kula K (2005) Fracture strength of ceramic bracket tie wings subjected to tension. Angle Orthod 75:95–100. https://doi.org/10.1043/0003-3219(2005)075〈0095:FSOCBT〉2.0.CO;2 15747822 10.1043/0003-3219(2005)075<0095:FSOCBT>2.0.CO;2

[44] Matek W, Muhs D, Wittel H (1986) Roloff/Matek Maschinenelemente, 10th edn. Vieweg & Sohn Verlagsgesellschaft mbH, Braunschweig

[45] Lindauer SJ, Macon CR, Browning H et al (1994) Ceramic bracket fracture resistance to second order arch wire activations. Am J Orthod Dentofacial Orthop 106:481–486. 10.1016/S0889-5406(94)70070-2 7977188 10.1016/S0889-5406(94)70070-2

[46] Guignone BC, Silva LK, Soares RV et al (2015) Color stability of ceramic brackets immersed in potentially staining solutions. Dental Press J Orthod 20:32–38. 10.1590/2176-9451.20.4.032-038.oar 26352842 10.1590/2176-9451.20.4.032-038.oar PMC4593527

[47] Braga de Oliveira C, Maia LG, Santos-Pinto A, Gandini Júnior LG (2014) In vitro study of color stability of polycrystalline and monocrystalline ceramic brackets. Dental Press J Orthod 19:114–121. 10.1590/2176-9451.19.4.114-121.oar 10.1590/2176-9451.19.4.114-121.oar PMC4296644 25279530

[48] Bishara SE, Fehr DE (1997) Ceramic brackets: something old, something new, a review. Semin Orthod 3:178–188. 10.1016/s1073-8746(97)80068-0 9573879 10.1016/s1073-8746(97)80068-0

[49] de Mendonça MR, Fabre AF, Goiatto MC et al (2011) Spectrophotometric evaluation of color changes of esthetic brackets stored in potentially staining solutions. RPG Rev Pós Grad 18:20–27

[50] Ruyter IE, Nilner K, Möller B (1987) Color stability of dental composite resin materials for crown and bridge veneers. Dent Mater 3:246–251. 10.1016/S0109-5641(87)80081-7 3479360 10.1016/S0109-5641(87)80081-7

[51] Akyalcin S, Rykiss J, Rody WJ, Wiltshire WA (2012) Digital analysis of staining properties of clear aesthetic brackets. J Orthod 39:170–175. 10.1179/1465312512Z.00000000024 22984101 10.1179/1465312512Z.00000000024

[52] Wriedt S, Schepke U, Wehrbein H (2007) The discoloring effects of food on the color stability of esthetic brackets—an in-vitro study. J Orofac Orthop 68:308–320. 10.1007/S00056-007-0640-2 17639279 10.1007/s00056-007-0640-2

[53] Schneevoigt R, Haase A, Eckardt VL et al (1999) Laboratory analysis of superelastic NiTi compression springs. Med Eng Phys 21:119–125. 10.1016/s1350-4533(99)00034-x 10426512 10.1016/s1350-4533(99)00034-x

[54] Ardeshna A, Chavan K, Prakasam A et al (2022) Effectiveness of different sterilization methods on clinical orthodontic materials. J Indian Orthod Soc. 10.1177/03015742221109026

[55] Vivek Aithal PR, Akshai Shetty KR, Dinesh MR et al (2019) In vitro evaluation of microbial contamination and the disinfecting efficacy of chlorhexidine on orthodontic brackets. Prog Orthod. 10.1186/s40510-019-0270-4 31041551 10.1186/s40510-019-0270-4 PMC6491528

